# Predictors for the prescription of pharmacological prophylaxis for venous thromboembolism during hospitalization in Internal Medicine: a sub-analysis of the FADOI-NoTEVole study

**DOI:** 10.1007/s11739-024-03770-w

**Published:** 2024-09-27

**Authors:** Alessia Abenante, Alessandro Squizzato, Lorenza Bertù, Dimitriy Arioli, Roberta Buso, Davide Carrara, Tiziana Ciarambino, Francesco Dentali

**Affiliations:** 1 Internal Medicine Department, ASST Sette Laghi, Varese, Italy; 2 Research Center on Thromboembolic Disorders and Antithrombotic Therapies, University of Insubria, Varese-Como, Italy; 3 Internal Medicine and Critical Area, AUO Modena, Modena, Italy; 4 Internal Medicine Department, University Hospital of Ca’ Foncello, Treviso, Italy; 5 Internal Medicine Unit, Hospital of Versilia, AUSL Toscana Nord-Ovest, Pisa, Italy; 6 Internal Medicine Department, Hospital of Marciabise, ASL Caserta, Caserta, Italy

**Keywords:** Thromboprophylaxis, Risk assessment models, Hospitalization, Internal medicine

## Abstract

Patients hospitalized in Internal Medicine Units (IMUs) may frequently experience both an increased risk for thrombosis and bleeding. The use of risk assessment models (RAMs) could aid their management. We present a post-hoc analysis of the FADOI-NoTEVole study, an observational, retrospective, multi-center study conducted in 38 Italian IMUs. The primary aim of the study was to evaluate the predictors associated with the prescription of thromboprophylaxis during hospitalization. The secondary objective was to evaluate RAMs adherence. Univariate analyses were conducted as preliminary evaluations of the variables associated with prescribing pharmacological thromboprophylaxis during hospital stay. The final multivariable logistic model was obtained by a stepwise selection method, using 0.05 as the significance level for entering an effect into the model. Thromboprophylaxis was then correlated with the RAMs and the number of predictors found in the multivariate analysis. Thromboprophylaxis was prescribed to 927 out of 1387 (66.8%) patients with a Padua Prediction score (PPS) ≥ 4. Remarkably, 397 in 1230 (32.3%) patients with both PPS ≥ 4 and an IMPROVE bleeding risk score (IBS) < 7 did not receive it. The prescription of thromboprophylaxis mostly correlated with reduced mobility (OR 2.31; 95% CI 1.90–2.81), ischemic stroke (OR 2.38; 95% CI 1.34–2.91), history of previous thrombosis (OR 2.46; 95% CI 1.49–4.07), and the presence of a central venous catheter (OR 3.00; 95% CI 1.99–4.54). The bleeding risk assessment using the IBS did not appear to impact physicians’ decisions. Our analysis provides insight into how indications for thromboprophylaxis were determined, highlighting the difficulties faced by physicians with patients admitted to IMUs.

**Supplementary Information:**

The online version contains supplementary material available at 10.1007/s11739-024-03770-w.

## Introduction

Venous thromboembolism (VTE) is a serious complication in hospitalized patients. The incidence of asymptomatic and symptomatic deep vein thrombosis (DVT) without pharmacological thromboprophylaxis is about 10 to 40% among patients admitted for medical issues [1, 2]. Randomized-controlled trials and meta-analyses showed that pharmacological prophylaxis significantly reduced the risk of VTE compared to placebo in medically ill inpatients [3, 4]. Patients hospitalized in Internal Medicine Units (IMUs) may frequently experience an increased bleeding risk due to coexistent diseases and treatments with potential drug-drug interactions. In these patients, the use of risk assessment models (RAMs) for predicting VTE and bleeding events is recommended by contemporary guidelines [5, 6]. The most validated RAMs to discriminate medical inpatients at higher risk for VTE are the Padua Prediction score (PPS) and the IMPROVE VTE score [7, 8]. The IMPROVE bleeding score (IBS) is suggested for identifying medical inpatients with an elevated bleeding risk [9]. These RAMs are not regularly used, as solid evidence of an effective positive impact on clinical practice is still lacking [10]. The use of pharmacological thromboprophylaxis after discharge and the factors associated with its use have been evaluated in a large Italian multicenter retrospective study, the FADOI-NoTEVole study [11]. The aim of this paper is to describe the predictors of pharmacological thromboprophylaxis among Italian physicians during IMUs stays of medical patients in a post-hoc analysis of the FADOI-NoTEVole study.

## Methods

### Study design

The FADOI-NoTEVole study was an observational, retrospective, multi-center study conducted in 38 Italian IMUs (either standard or short-stay units) from September to December 2017. The study protocol was approved by the Medical Ethics Committee (CER Umbria) of Perugia, Italy, and reported to the Ethics Committees of the other participating sites. The study was conducted according to the Declaration of Helsinki (Fortaleza 2013 version). The study was coordinated by the Scientific Society of Internal Medicine (FADOI, i.e., Federazione delle Associazioni dei Dirigenti Ospedalieri Internisti).

### Objective

The primary aim of the study is to evaluate the rate of pharmacological prophylaxis for VTE, and the predictors associated with the prescription of pharmacological prophylaxis during the hospital stay. The secondary aim is to evaluate RAMs’ adherence.

### Inclusion criteria

The inclusion criteria were described in the main study paper [11]. Briefly, all the consecutive medically ill patients admitted for any cause and discharged alive from any participating IMU were considered. Patients with a short life expectancy (less than 3 months) who were transferred to the nursing home, or the palliative care service were excluded. Patients on therapeutic doses of oral or parenteral anticoagulants at admission were excluded.

### Data collection

Data collection was described in the main study paper [11]. The available information from the hospital charts is listed as follows:

- Age, gender, body mass index (BMI).
- Medical history (history of VTE, history of major bleeding according to the International Society of Thrombosis and Haemostasis (ISTH) [12], bleeding events occurred less than 3 months earlier, bed rest, lower limb paralysis, thrombophilia, central venous catheter, trauma, or surgery within 1 month before the hospitalization, alcohol abuse, risk of falling in the previous 6 months, availability of a caregiver).
- Laboratory analysis (hemoglobin, platelet count, creatinine, International Normalized Ratio, activated Partial Thromboplastin Time).
- Acute diseases at hospital admission and concomitant diseases.
- Drugs (anti-inflammatory medications, anticoagulants, hormone therapy, antibiotics, oxygen therapy).
- RAMs for the risk of venous thrombosis and bleeding (Padua Prediction Score and the IMPROVE Bleeding Score).
- Clinical events occurring during hospital stay (major bleeding, VTE, other major cardiovascular events).
- Pharmacological prophylaxis for VTE during hospital stays (type and duration throughout the hospital stay, type and expected duration at discharge).

The enrolled cohort was divided into two groups for the main analyses: those who used pharmacological thromboprophylaxis and those who did not. For the secondary analyses, enrolled patients were divided between those with a Padua Prediction score < 4 or ≥ 4, and those with an IMPROVE bleeding score < 7 or ≥ 7, analyzing their characteristics.

### Statistical analysis

The frequency of pharmacological thromboprophylaxis was presented with a corresponding 95% confidence interval (CI) corrected for continuity correction. All categorical variables were expressed as the absolute number and percentage, and continuous variables as mean and standard deviation or median and interquartile range if not normally distributed.

Odds ratios (OR) and 95% confidence intervals were reported with their respective two-tailed probability values. A *p*-value of < 0.05 was considered statistically significant. A set of univariate analyses were carried out as preliminary evaluations of the variables associated with the use of pharmacological thromboprophylaxis during hospital stay. The final multivariable logistic model was obtained by a stepwise selection method, using 0.05 as the significance level for entering an effect into the model. The same significance level (*p*-value < 0.05) was required for a variable to stay in the model. The use of prophylaxis was then correlated with the RAMs and with the number of predictors shown in the multivariate analysis. All statistical analyses were performed using SAS ODA (version 9.4, SAS Institute, Cary, North Carolina, United States).

## Results

### Baseline characteristics

**Fig. 1:**
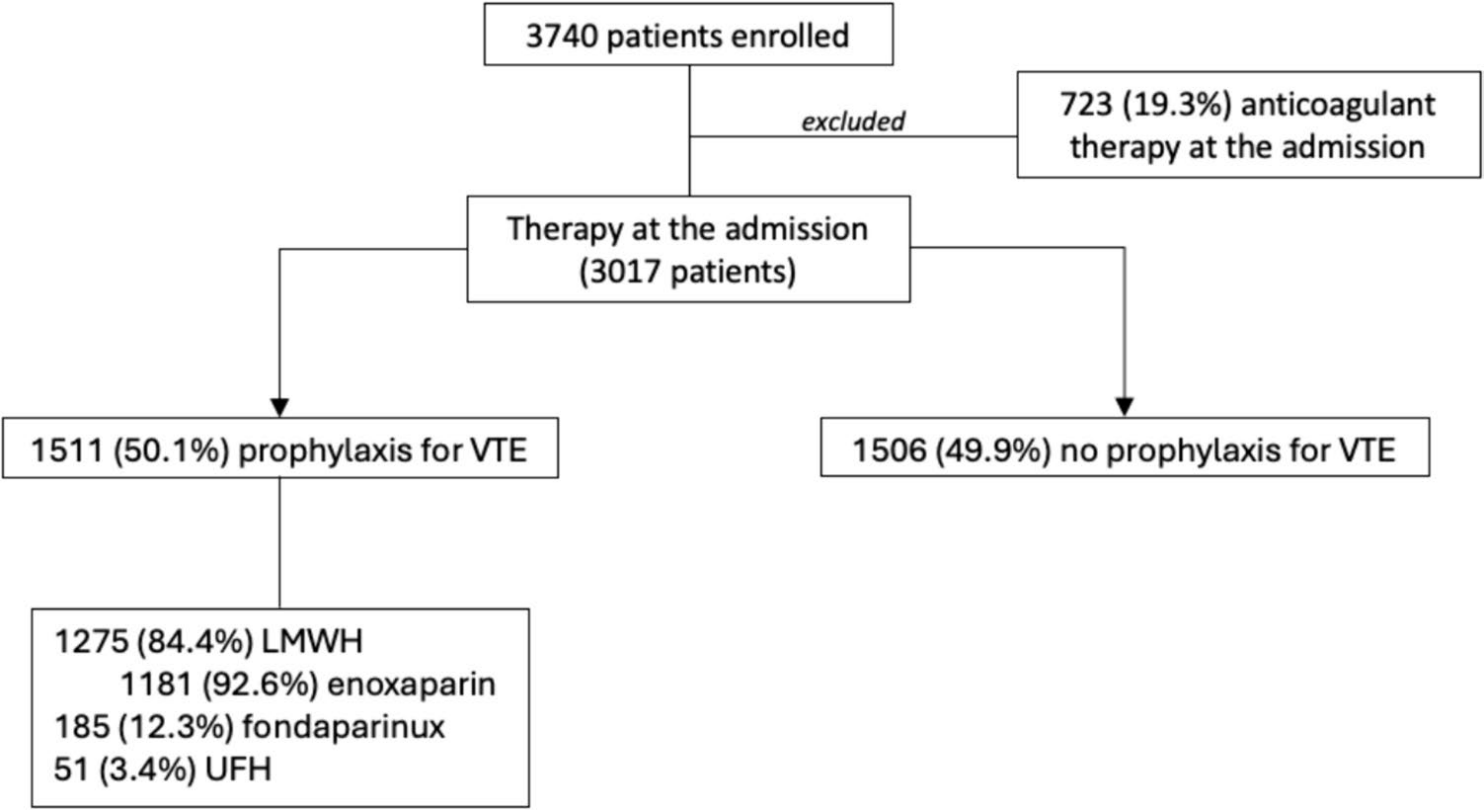
Flowchart of cohort selection (*VTE* venous thromboembolism, *LMWH* low molecular weight heparin, *UFH* unfractionated heparin)

The NoTEVole study enrolled 3740 patients with a mean age of 74.1 years. Our analysis excluded 723 patients (19.3%) on anticoagulant therapy at admission, considering an overall population of 3017 patients. Among them, 1511 (50.1%) were treated with VTE prophylaxis during the hospital stay, and 1506 (49.9%) were not. Low molecular weight heparin was the most used pharmacological thromboprophylaxis (84.4%), mainly enoxaparin (78.2%). The selection of the analyzed cohort is detailed in Fig. 1.

Almost all patients (97.6%) were hospitalized in a standard IMU with a median hospital stay of 9 days, of them, 487 patients (16.1%) were hospitalized for less than 5 days. The 54.3% of the population was over 75 years old, with an equal distribution of gender (female 50.2%), and 16% had a BMI > 30 kg/m^2^. Considering the main diagnosis at admission, 35.3% of patients had an acute infection, 20.3% had heart failure, 7.6% had severe renal impairment (GFR < 30 ml/min), and 6.3% had active cancer. Exploring the thrombotic risk, 46% of the population had a Padua Prediction score ≥ 4, and 3.3% had a history of VTE. The main baseline characteristics are detailed in Table 1.

**Table 1:** Baseline characteristics and main diagnosis at admission

	General cohort	Prophylaxis during hospitalization		Wald *p*-value*
		Yes	No	
N of patients	3017	1511	1506	
Age (median IQR)	77 (65–85)	80 (71–87)	71 (57–82)	< 0.01
Female (%)	1514 (50.2)	798 (52.8)	716 (47.5)	< 0.01
BMI > 30 (%)	482 (16.0)	255 (52.9)	227 (47.1)	0.18
GFR ≥ 60 ml/min (%)	2362 (78.3)	1147 (48.6)	1215 (51.4)	< 0.01
GFR 30–59 ml/min (%)	426 (14.1)	239 (56.1)	187 (43.9)	
GFR < 30 ml/min (%)	229 (7.6)	125 (54.6)	104 (45.4)	
Active cancer (%)	350 (11.6)	200 (57.1)	150 (42.9)	< 0.01
History of major bleeding (%)	184 (6.1)	88 (47.8)	96 (52.2)	0.60
Heart failure (%)	612 (20.3)	415 (67.9)	197 (32.1)	< 0.01
Acute infection (%)	1065 (35.3)	660 (62.0)	405 (38.0)	< 0.01
Ischemic stroke (%)	138 (4.5)	88 (63.8)	50 (36.2)	< 0.01
CVC (%)	161 (5.3)	123 (76.4)	38 (23.6)	< 0.01
History of VTE (%)	99 (3.3)	74 (74.8)	25 (25.2)	< 0.01
COPD (%)	476 (15.8)	299 (62.8)	177 (37.2)	< 0.01
Reduced mobility** (%)	782 (25.9)	552 (70.6)	230 (29.4)	< 0.01
Antiplatelet therapy (%)	1044 (34.6)	589 (56.4)	455 (43.6)	< 0.01
Hb g/dl (median IQR)	11.6 (10.0–13.1)	11.4 (10.0–12.8)	11.9 (10.0–13.4)	
PLT*10^9^/mm^3^ (median IQR)	228 (172–297)	235 (179–314)	222 (167–283)	

*Wald *p*-value is derived from the univariate analysis of the baseline cohort’s characteristics

**reduced mobility ≥ 7 days before hospitalization

*IQR* interquartile range, *BMI* body mass index, *GFR* glomerular filtration rate, *CVC* central venous catheter, *COPD* chronic obstruction pulmonary disease, *Hb* hemoglobin, *PLT* platelet count, *VTE* venous thromboembolism

### VTE and bleeding risk stratification with RAMs and the use of thromboprophylaxis

**Fig. 2:**
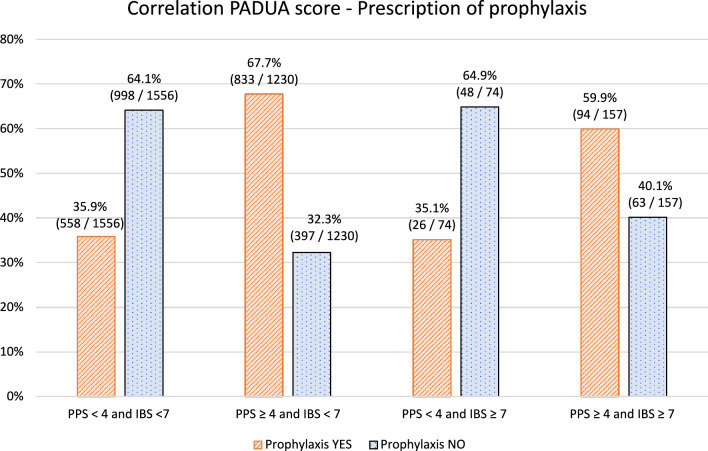
Application of the RAMs and prescription of thromboprophylaxis (*PPS* Padua prediction score, *IBS* improve bleeding score)

Thromboprophylaxis was prescribed to 927 out of 1387 (66.8%) patients with a Padua Prediction score of 4 or higher, while 460 (33.2%) did not receive it (see Table 2 and Fig. 2). Regarding the hemorrhagic risk, 213 patients (7.7%) had an IMPROVE bleeding score of 7 or more, and 120 (51.9%) had thromboprophylaxis prescribed. Moreover, 397 out of 1230 (32.3%) patients with both a high thrombotic risk (PPS ≥ 4) and a low bleeding risk (IBS < 7) did not receive thromboprophylaxis. On the other hand, in the population with a low thrombotic risk (PPS < 4) and a high hemorrhagic risk (IBS ≥ 7), 26 out of 74 patients (35.1%) still received pharmacological prophylaxis.

**Table 2:** Application of the RAMS and prescription of thromboprophylaxis

RAMs	General population	Prophylaxis			
	3017	Yes (1511)	No (1506)	OR	CI 95%
IMPROVE Bleeding ≥ 7 (n%)	231 (7.7)	120 (51.9)	111 (48.1)	1.08	0.83–1.42
Padua Prediction score ≥ 4 (n%)	1387 (46.0)	927 (66.8)	460 (33.2)	3.61	3.10–4.20
PPS < 4 and IBS < 7 (n%)	1556 (51.6)	558 (36.9)	998 (66.3)	1.0	reference
PPS ≥ 4 and IBS < 7 (n%)	1230 (40.8)	833 (67.7)	397 (32.2)	3.7	3.2–4.4
PPS < 4 and IBS ≥ 7 (n%)	74 (2.5)	26 (35.1)	48 (64.9)	1.0	0.6–1.6
PPS ≥ 4 and IBS ≥ 7 (n%)	157 (5.2)	94 (59.9)	63 (40.1)	2.7	1.9–3.7

RAMs risk model assessments, PPS Padua prediction score, IBS improve bleeding score, OR odds ratio, CI 95% confidence interval of 95%

### Correlation between risk factors and prescription of thromboprophylaxis

Patients who received pharmacological thromboprophylaxis were older (OR 1.04; 95% CI 1.03–1.04) and with a worse renal function (GFR < 30 ml/min, OR 1.27; 95% CI 0.97–1.67) compared to patients who did not receive it. The following variables were identified as candidates for the multivariable analysis: age, sex, reduced mobility, the presence of a central venous catheter, kidney impairment, active cancer, heart failure, ischemic stroke, infection, hemoglobin < 10 g/dl, platelet ≥ 70.000/mm^3^, previous TEV, corticosteroid’s use, and previous major bleedings.

**Fig. 3:**
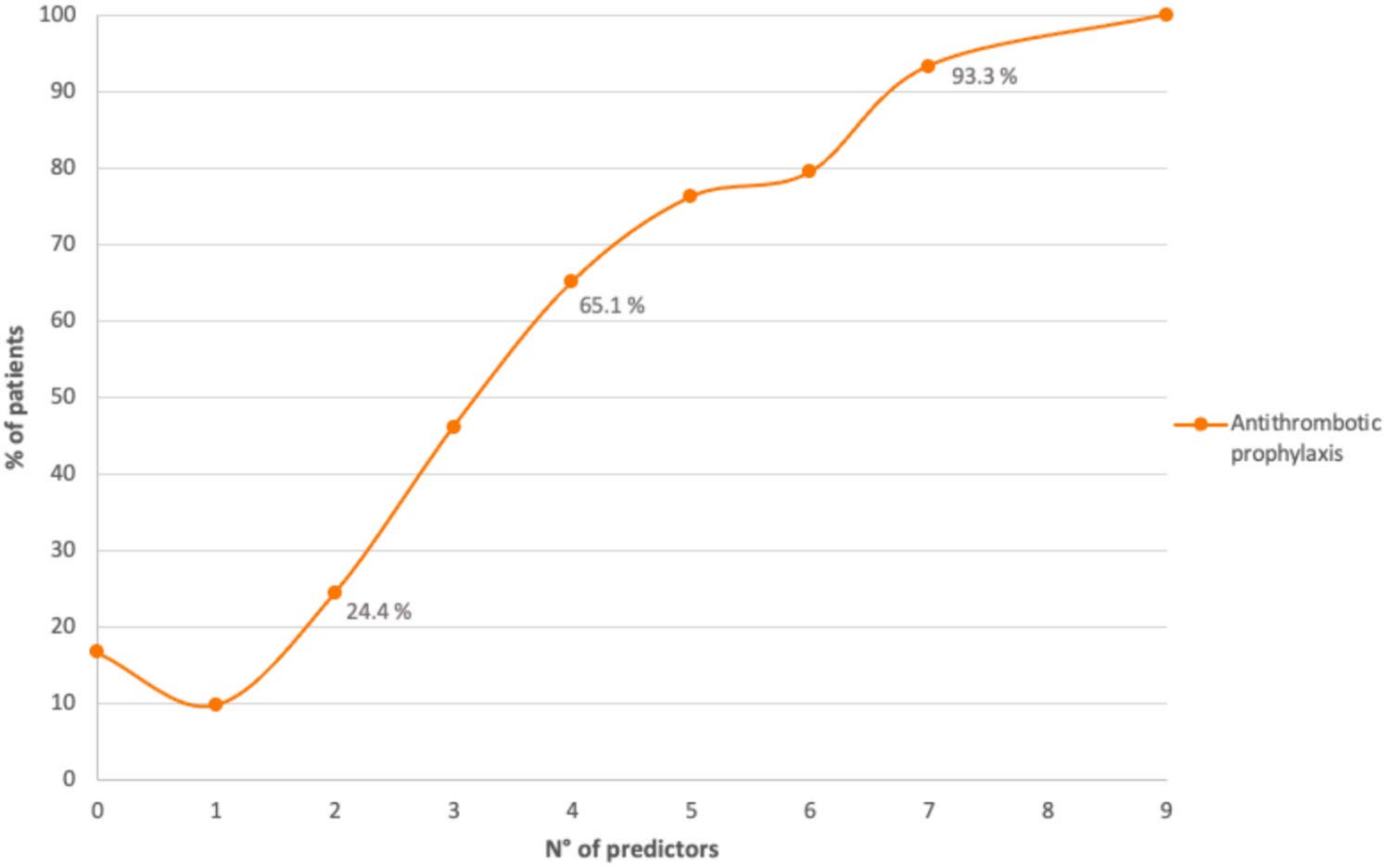
Correlation between the number of predictors of thromboprophylaxis prescription from the multivariate analysis and the actual prescription of thromboprophylaxis (*VTE* venous thromboembolism)

At the multivariate analysis, reduced mobility (OR 2.31; 95% CI 1.90–2.81), ischemic stroke (OR 2.38; 95% CI 1.34–2.91), previous VTE (OR 2.46; 95% CI 1.49–4.07), infection at admission (OR 2.22; 95% CI 1.87–2.64), presence of a central venous catheter (OR 3.00; 95% CI 1.99–4.54) and platelet count ≥ 70.000/mm^3^ (OR 3.11; 95% CI 1.81–5.34) resulted significantly associated with thromboprophylaxis’ prescription while the history of previous bleeding was associated with a lower rate of prescription (OR 0.61; 95% CI 0.44–0.86) (see Table 3). Also, the use of pharmacological prophylaxis progressively increased with the number of predictors, rising from 9.8% with a single item to 100% in patients with 9 items (Fig. 3). Detailed data are reported in *Supplementary S2.*

**Table 3:** Association between baseline variables and thromboprophylaxis during hospitalization, multivariate analysis

Associated variables	OR	95% CI
Reduced mobility^⊺^	2.31	1.90–2.81
Age (continuous)	1.03	1.02–1.04
CVC	3.00	1.99–4.54
Cancer	2.18	1.39–2.29
Heart failure	2.18	1.76–2.69
Ischemic stroke	2.38	1.34–2.91
Acute infection	2.22	1.87–2.64
Hb < 10 g/dl	1.26	1.04–1.53
PLT ≥ 70*10^9^/mm^3^	3.11	1.81–5.34
Previous VTE	2.46	1.49–4.07
Previous major bleedings	0.61	0.44–0.86

*OR* odds ratio, *BMI* body mass index, *GFR* glomerular filtration rate, *CVC* central venous catheter, *COPD* chronic obstruction pulmonary disease, *Hb* hemoglobin, *PLT* platelet count, *VTE* venous thromboembolism

⊺reduced mobility ≥ 7 days

## Discussion

This subanalysis of the FADOI-NoTEVole study provides detailed insight into Italian physicians’ practice regarding the use of pharmacological thromboprophylaxis in IMU inpatients. No specific guidelines were indicated in the study’s protocol regarding the prescription of prophylaxis; the choice was completely left to the physician.

Pharmacological thromboprophylaxis was prescribed in about half of the 3017 patients admitted to 38 Italian IMUs without prior anticoagulation. According to the PPS, almost 50% of patients were considered at high risk of VTE and, according to the IBS, less than 8% were considered at high risk for bleeding. Although not specifically requested by the FADOI-NoTEVole study protocol, the adoption of pharmacological thromboprophylaxis during hospitalization was only partially influenced by the VTE and bleeding RAMs recommended by current guidelines [5, 6]. Indeed, only 66.8% of the patients classified as high risk for VTE, according to the PPS, received thromboprophylaxis. Additionally, the assessment of bleeding risk using the IBS did not appear to impact physicians’ decisions. Interestingly, several other features composing PPS, IBS, and other RAMs [7, 8] were associated with a different use of pharmacological prophylaxis. These included the presence of a CVC, a previous VTE event, the diagnosis of ischemic stroke, perceived reduced mobility, and a platelet count not lower than 70.000/mm^3^ at the time of admission. On the other hand, as expected, a recent former major bleeding event led to a lower prescription of pharmacological thromboprophylaxis. Many studies have evaluated the role of potential risk factors for VTE and bleeding in patients hospitalized in non-surgical settings. Based on the findings of the IMPROVE VTE score and Padua Prediction score studies, reduced mobility and previous VTE were among the factors most strongly associated with the development of VTE [7, 8]. Venous thromboembolism, including DVT and pulmonary embolism (PE), is a frequent complication in bedridden patients with acute ischemic stroke [13]. Therefore, preventing venous thromboembolic complications of stroke could help reduce the burden of stroke-related disabilities. Randomized-controlled trials and a meta-analysis have clearly demonstrated that the use of antithrombotic prophylaxis with low molecular weight heparin in these patients is associated with a reduced risk of DVT and PE [14]. However, pharmacologic thromboprophylaxis does not affect mortality and is associated with a small but not negligible increased risk of bleeding, leaving some uncertainty regarding its use in this setting [15]. Central venous catheters are increasingly used in clinical practice and have a non-negligible incidence of catheter-related VTE, which is often asymptomatic. Recent studies have failed to find a significant reduction in symptomatic VTE with the use of antithrombotic prophylaxis [16, 17]. Furthermore, the IMPROVE registry indicates that the presence of a central venous catheter results in a significantly increased risk of bleeding [9].

In previous studies, the use of pharmacological thromboprophylaxis in hospitalized non-surgical patients was highly heterogeneous. In the Endorse study, a multinational cross-sectional study designed to assess the prevalence of VTE risk in the acute hospital care setting, the use of pharmacological thromboprophylaxis was generally low, although the risk of VTE was not negligible [18]. Conversely, in a large prospective Italian cohort study*,* pharmacological thromboprophylaxis was implemented in more than 80% of patients admitted to an IMU with a PPS equal to or above 4 [19].

Several randomized controlled trials and meta-analyses have clearly shown the efficacy of pharmacological thromboprophylaxis compared to placebo in reducing the risk of VTE in non-surgical patients admitted to the hospital [4, 20, 21]. However, this is associated with an increased risk of bleeding and, in general, it has no effect on reducing all-cause mortality, questioning the real importance of this treatment in medical settings. Thus, tools to assess individual risk of VTE and bleeding may be useful in this setting.

In the last few years, many RAMs evaluating these risks have been developed and validated. However, poor evidence is available assessing their real impact in improving the clinical outcomes of these patients [10]. In a small single-center, prospective, quasi-randomized study, the adoption of the Padua Prediction Score was associated with a 50% reduction in the incidence of VTE (i.e., symptomatic and asymptomatic, mainly distal VTE) compared with clinical judgment, with no differences in terms of bleeding and death from all-cause [22]. In a large RCT, employing a strategy including an electronic alert about the thrombotic risk (assessed through a risk score) versus no alert, greatly reduced the occurrence of symptomatic VTE with no increase in the bleeding rate in hospitalized patients [23].

Our study has some limitations. First, the retrospective design is *per definition* at risk of bias. Data are acquired from clinical records with the risk of suboptimal reporting. This may lead to possible bias in the post-hoc assessment of the RAMs, but the distribution of the thrombotic and bleeding risk appeared to be in line with the previous literature. Second, no information was available about the use of mechanical prophylaxis (i.e., intermittent pneumatic compression and elastic stockings) and early mobilization. Third, the FADOI-NoTEVole study included only patients discharged alive and excluded patients with a life expectancy of less than three months who were transferred to the nursing home, or the palliative care service: this resulted in a lack of data regarding mortality rates. Also, while the sample size is reasonable for analyzing potential predictors for prescribing thromboprophylaxis, it may still be small for some variables, leaving a margin of uncertainty. Lastly, since this is a retrospective observational study, we do not know whether a positive D-dimer value may have sometimes guided the physician’s choice, as previous studies correlated D-dimer with venous thrombosis [24]. Unfortunately, this data was not collected, and thus, it was not used in the study analyses.

In conclusion, our analysis showed some factors that may influence physicians’ prescription of pharmacological thromboprophylaxis, underscoring the daily challenges in assessing the appropriateness of pharmacological thromboprophylaxis in IMU’s patients. Single risk factors for VTE and bleeding appear to modify clinicians’ behavior. Of note, PPS seems to be only a partial driver of the use of pharmacological prophylaxis, and IBS does not appear to modify clinicians’ approach at all.

## Supplementary Information

Below is the link to the electronic supplementary material.

Supplementary file1 (DOCX 23 KB)

## Data Availability

Not applicable.
