# Supplementary material for: Predictors for the prescription of pharmacological prophylaxis for venous thromboembolism during hospitalization in Internal Medicine: a sub-analysis of the FADOI-NoTEVole study
Source: Intern Emerg Med. 2024 Sep 27;20(1):151–8. doi: 10.1007/s11739-024-03770-w (PMC11794402; doi:10.1007/s11739-024-03770-w)
Supplement: Supplementary file 1 — Supplementary file1 (DOCX 23 KB) [file 11739_2024_3770_MOESM1_ESM.docx]

**SUPPLEMENTARY MATERIAL**

|  | | **Univariate** | | **Wald**  **p-value** | **Multivariate*** | |
| --- | --- | --- | --- | --- | --- | --- |
|  |  | **OR** | **95%CI** |  | **OR** | **95%CI** |
| **Age (continuous)** |  | 1.04 | 1.03-1.04 | <0.0001 | 1.03 | 1.02-1.04 |
| **Sex** | Female | 1.0 | reference | 0.004 |  |  |
|  | Male | 0.81 | 0.70-0.93 |  |  |  |
| **BMI** | <30 | 1.0 | reference | 0.18 |  |  |
|  | ≥30 | 1.14 | 0.94-1.39 |  |  |  |
| **Reduced mobility** | No | 1.0 | reference | <0.0001 | 1.0 | reference |
|  | Yes | 3.19 | 2.68-3.81 |  | 2.31 | 1.90-2.81 |
| **Known thrombophilia** | No | 1.0 | reference | 0.70 |  |  |
|  | Yes | 1.16 | 0.54-2.53 |  |  |  |
| **CVC** | No | 1.0 | reference | <0.0001 | 1.0 | reference |
|  | Yes | 3.42 | 2.36-4.96 |  | 3.00 | 1.99-4.54 |
| **CKD**  **GFR ml/min** | No | 1.0 | reference | 0.006 |  |  |
|  | 30-59 | 1.35 | 1.10-1.67 |  |  |  |
|  | <30 | 1.27 | 0.97-1.67 |  |  |  |
| **Cancer** | No | 1.0 | reference | 0.005 | 1.0 | reference |
|  | Yes | 1.38 | 1.10-1.73 |  | 2.18 | 1.39-2.29 |
| **Heart failure** | No | 1.0 | reference | <0.0001 | 1.0 | reference |
|  | Yes | 2.52 | 2.09-3.04 |  | 2.18 | 1.76-2.69 |
| **Ischemic stroke** | No | 1.0 | reference | 0.001 | 1.0 | reference |
|  | Yes | 1.80 | 1.26-2.57 |  | 2.38 | 1.34-2.91 |
| **Infection** | No | 1.0 | reference | <0.0001 | 1.0 | reference |
|  | Yes | 2.11 | 1.81-2.46 |  | 2.22 | 1.87-2.64 |
| **Hb<10 g/dl** | No | 1.0 | reference | 0.82 | 1.0 | reference |
|  | Yes | 1.02 | 0.86-1.20 |  | 1.26 | 1.04-1.53 |
| **PLT ≥70*10^9^/mm^3^** | No | 1.0 | reference | <0.0001 | 1.0 | reference |
|  | Yes | 2.87 | 1.77-4.65 |  | 3.11 | 1.81-5.34 |
| **Use of corticosteroids** | No | 1.0 | reference | 0.004 |  |  |
|  | Yes | 1.35 | 1.10-1.66 |  |  |  |
| **Previous VTE** | No | 1.0 | reference | <0.0001 | 1.0 | reference |
|  | Yes | 3.05 | 1.93-4.82 |  | 2.46 | 1.49-4.07 |
| **Previous major bleedings** | No | 1.0 | reference | 0.60 | 1.0 | reference |
|  | Yes | 0.92 | 0.69-1.25 |  | 0.61 | 0.44-0.86 |
| BMI: body mass index, GFR: glomerular filtration rate, CVC: central venous catheter, COPD: chronic obstruction pulmonary disease, Hb: hemoglobin, PLT: platelet count, VTE: venous thromboembolism | | | | | | |

***Supplementary S1.*** *Association between baseline variables and thromboprophylaxis during hospitalization, univariate and multivariate analysis.*

| **N of predictors**  **for VTE** | **Thromboprophylaxis** | |
| --- | --- | --- |
|  | **NO (n%)** | **YES (n%)** |
| 0 | 83,3 | 16,7 |
| 1 | 90,2 | 9,8 |
| 2 | 75,6 | 24,4 |
| 3 | 53,9 | 46,1 |
| 4 | 34,9 | 65,1 |
| 5 | 23,8 | 76,2 |
| 6 | 20,6 | 79,4 |
| 7 | 6,7 | 93,3 |
| 9 | 0 | 100 |

***Supplementary S2.*** *Correlation between the number of* ***predictors for prescription*** *(from Table 2) and the prescription of thromboprophylaxis.*
